# Changes in apoptotic microRNA and mRNA expression profiling in *Caenorhabditis elegans* during the Shenzhou-8 mission

**DOI:** 10.1093/jrr/rrv050

**Published:** 2015-08-17

**Authors:** Ying Gao, Shuai Li, Dan Xu, Junjun Wang, Yeqing Sun

**Affiliations:** Institute of Environmental Systems Biology, College of Environmental Science and Engineering, Dalian Maritime University, Linghai Road 1, Dalian 116026, China

**Keywords:** apoptosis, spaceflight, microRNA, core apoptotic gene

## Abstract

Radiation and microgravity exposure have been proven to induce abnormal apoptosis in microRNA (miRNA) and mRNA expression, but whether space conditions, including radiation and microgravity, activate miRNAs to regulate the apoptosis is undetermined. For that purpose, we investigated miRNome and mRNA expression in the *ced-1 Caenorhabditis elegans* mutant vs the wild-type, both of which underwent spaceflight, spaceflight 1g-centrifuge control and ground control conditions during the Shenzhou-8 mission. Results showed that no morphological changes in the worms were detected, but differential miRNA expression increased from 43 (ground control condition) to 57 and 91 in spaceflight and spaceflight control conditions, respectively. Microgravity altered miRNA expression profiling by decreasing the number and significance of differentially expressed miRNA compared with 1 g incubation during spaceflight. Alterations in the miRNAs were involved in alterations in apoptosis, neurogenesis larval development, ATP metabolism and GTPase-mediated signal transduction. Among these, 17 altered miRNAs potentially involved in apoptosis were screened and showed obviously different expression signatures between space conditions. By integrated analysis of miRNA and mRNA, miR-797 and miR-81 may be involved in apoptosis by targeting the genes *ced-10* and both *drp-1* and *hsp-1,* respectively. Compared with ground condition, space conditions regulated apoptosis though a different manner on transcription, by altering expression of seven core apoptotic genes in spaceflight condition, and eight in spaceflight control condition. Results indicate that, miRNA of *Caenorhabditis elegans* probably regulates apoptotic gene expression in response to space environmental stress, and shows different behavior under microgravity condition compared with 1 g condition in the presence of space radiation.

## INTRODUCTION

The space environment, which is characterized by intense radiation, microgravity and other factors, profoundly differs from the environment on Earth [1, 2]. Space radiation and microgravity are recognized as the primary and inevitable risk factors for humans traveling in space [3–6]. Previous studies report that the damage to living organisms occurring during spaceflight is often concomitant with abnormal apoptosis at cellular and/or molecular levels [7–10]. Apoptosis is vital for development, tissue formation and the survival of multicellular organisms, because it exerts protective effects against inflammatory responses or autoimmune diseases in tissues by eliminating abnormal or unnecessary cells [11] and maintaining genomic stability during DNA damage response (DDR) [12, 13]. Although ground-based studies report a variety of modality induced by ionizing radiation (IR) or modeled microgravity (MMG) on apoptosis [14–18], the modality by which microgravity incubation affects IR-induced apoptosis remains controversial [19–22].

MicroRNAs (miRNAs), a class of small non-coding RNA, mediate post-transcriptional regulation of specific target mRNAs in various cellular processes [23]. Several studies have reported that IR induces changes in miRNA expression, *in vitro* and *in vivo*, according to cell type, radiation dose and linear energy transfer (LET) [24–26], and suggested that miRNA expression is regulated in DDR, including apoptosis [27]. Changes in miRNAs, e.g. miR-9, 150, also represent the post-transcriptional responses to microgravity using human lymphoblastoid cells [28, 29]. Girardi *et al.* found that eight miRNAs were deregulated by the combined action of radiation and MMG [24], suggesting that miRNA expression represents environmental specificity. While the response of miRNAs involved in apoptosis during spaceflight has not been studied yet, it should give important information about the risks of the exposure to space environment.

It is noteworthy that the core apoptotic pathway includes decision, execution, engulfment and DNA degradation [12]. Therefore, there are some limitations to studying integrated apoptosis alterations in an individual metazoan by observing the response of cellular material. *Caenorhabditis elegans* (*C. elegans*) is a well-characterized metazoan in the field of apoptosis study, and abundant research with *C. elegans* has established the conserved core apoptotic machinery under genetic control [12, 30]. *C. elegans* also has been used for several space biological studies and shown good tolerance during spaceflights [31, 32]. Research has shown that the transcriptional profiling of *C. elegans* could alter in response to spaceflight conditions [33]; mutant strains responded to space conditions in a different manner compared with wild-type *C. elegans* [34–36]. However, the checkpoints and physiological apoptosis in germ cells proceeded normally, both in the *ced-1* mutant and wild-type *C. elegan*s [31]. We have also investigated the mRNA and miRNA expression profiling by using diapause dauer larvae of *C. elegans*. Our previous results have shown changes in miRNA expression profiling in response to different space conditions [37], as well as in the expression of non-core apoptotic genes [38]. Given the apoptotic gene expression dynamics regulated by miRNAs [39, 40], miRNAs of *C. elegans* were speculated to regulate the cellular tolerance/resistance to apoptosis during spaceflight.

In the present study, we focus on the changes in apoptosis induced by space conditions at the post-transcriptional level, and the difference in post-transcriptional regulation between wild-type and an abnormal apoptotic mutant during spaceflight. For these purposes, we investigated the differences in miRNA expression profiling and in core apoptotic mRNA expression between *ced-1* mutant and wild-type *C. elegans* at the dauer stage. By using diapause dauer larvae, which present better resistance to reactive oxygen species (ROS) damage and genomic instability [41–43], we can avoid the influence of metagenesis and the different development stages during spaceflight. In *C. elegans*, CED-1 triggers a signaling pathway in phagocytic cells that promotes cell corpse engulfment, phagosome maturation and apoptotic cell degradation [44, 45]. Dysfunctional mutation of the *ced-1* gene produces weak defects but does not totally block cell engulfing [46], because *ced-1* functioning with *ced-6*-*ced-7* forms a partially redundant pathway with *ced-2*-*ced-5*-*ced-12* for controlling engulfment [12].

## MATERIALS AND METHODS

### Sample preparation and spaceflight experiments

The wild-type strain and *ced-1* (*n1995*) of *C. elegans* were obtained from the *Caenorhabditis* Genetics Center (Minneapolis, MN, USA). As described in our previous study [38], worms were maintained on solid nematode growth medium (NGM) [47] and approximately 10^5^ synchronized dauer larvae were separately loaded into static and 1g-centrifuge experimental containers (Table 1). During the 16.5-day Shenzhou-8 mission (1–17 November 2011), the environment was maintained at a temperature of 23 ± 0.5°C, and a relative humidity of 20.79–56.35%. Space radiation doses were measured at 1.92 mGy (static slot) and 2.27 mGy (centrifuge slot). The corresponding ground control conditions were performed in parallel at the Payload Integration Test Centre in Beijing two days later. Seven hours after landing, several worms were transfer to new NGM with *E. coli* OP50 for morphology observation (L4 stage) after ∼10 h [47], and others were collected and kept in liquid nitrogen for further studies.

**Table 1.** Experimental groups setting and indicated meaning

No.	Group	Treatment	Radiation^a^	Microgravity
1	Spaceflight	Spaceflight static slot	1.92 mGy	μg
2	Spaceflight control	Spaceflight 1g-centrifuge slot	2.27 mGy	1g
3	Ground control	Ground static slot	–	1g

^a^Space radiations dose was measured by the thermoluminescent detector during spaceflight.

### Total RNA isolation

As described in our previous study, about 2000 worms from each group were collected, and total RNA was isolated using Invitrogen^TM^ TRIzol (Invitrogen, Carlsbad, CA, USA) and the miRNeasy mini kit (Qiagen, Valencia, CA, USA), according to the manufacturer's instructions [38]. Quality and purity of the RNA preparations were assessed with the OD260/OD280 ratio and quantification of the ratios of 28S:18S ribosomal RNA using the NanoDrop 2000 (Thermo Fisher, Wilmington, DE, USA) and the GelDoc-ItTM 310 Imaging System (UVP, Cambridge, CA, EUA), respectively.

### miRNA expression analysis and target prediction

The NimbleGen Gene Expression Profiling service and miRCURY™ LNA Array microRNA Expression Profiling service were performed by KangChen Bio-tech Inc. (Shanghai, China) as previously described [38]. Differentially expressed miRNAs (>1.5-fold change and <0.67) and mRNAs (>2-fold change and <0.5) were identified through fold-change filtering. To predict the putative target genes of miRNA, computational analysis was performed with at least two combinations of MicroRNA (www.microrna.org/microrna/home.do), Microcosm Targets (Version 5, www.ebi.ac.uk/enright-srv/microcosm/htdocs/targets/v5/), PicTar (pictar.mdc-berlin.de/cgi-bin/new_PicTar_nematode.cgi?species = nematode) and Targetscan Worm 6.2 (www.targetscan.org/worm_52/). We performed a gene ontology (GO) analysis using the DAVID tool (david.abcc.ncifcrf.gov/) to identify which biological processes were significantly enriched among the target genes (*P* < 0.05).

### Apoptotic miRNA identification and anti-correlated analysis of miRNA and genes

To predict which *C. elegans* miRNAs were involved in the apoptosis process, we used the miRò tool (ferrolab.dmi.unict.it/miro/index.php) to screen human miRNAs involved in apoptosis by GO analysis, then compared the similarity of mature miRNA sequences between human and *C. elegans* according to Ibanez's study in order to obtain potential apoptotic miRNAs in *C. elegans* [48]*.* To identify the most likely targets, we integrated gene and miRNA expression data obtained for the same biological sample to screen the anti-correlated putative pairs of miRNA and mRNA.

### Verification of microarray data by qRT-PCR

The data of the mRNA and of the miRNA microarray were validated by quantitative real-time polymerase chain reaction (qRT-PCR). Total RNA independent from that used in microarrays was extracted from ∼2000 worms with Invitrogen^TM^ TRIzol (Invitrogen, Carlsbad, CA, USA). qRT-PCR was performed with the SuperScript® III Reverse Transcriptase (Invitrogen, Carlsbad, CA, USA) and SYBR® Green Master Mix (Applied Biosystems, Foster City, CA, USA) as described in previous studies [38]. The miRNA levels were adjusted to that of internal standard U6, and the results were statistically analyzed by the 2^−△△C^^t^ method.

## RESULTS

### Morphology observation

**Fig. 1.**
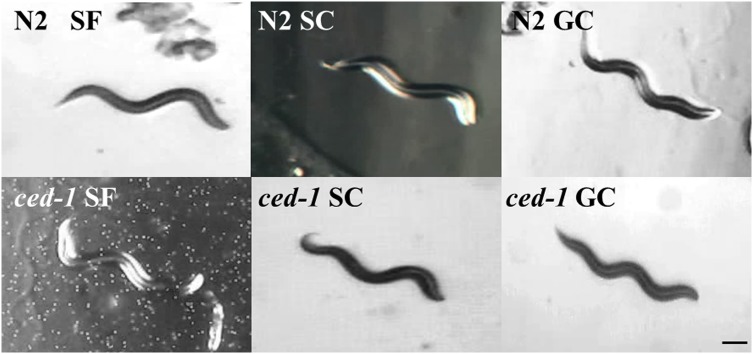
Image of L4 stage larva of *C. elegans* after spaceflight experiment. Scale bars represent 100 μm. N2, wild-type; *ced-1*, *ced-1* mutant strain. SF, spaceflight; SC, spaceflight control; GC, ground control. Due to malfunctions CCD device in the spaceflight landing scene, the images showed darker background brightness with white noise when checking “N2 SC” and “*ced-1* SF” worm.

As shown in Fig. 1, after a 16.5-day spaceflight and ∼10 h development on ground, dauer larvae could molt to the L4 stage. There were no obvious or microscopic morphological abnormalities observed in the wild-type or *ced-1* mutant worms.

### Analysis of miRNA expression profiling and target genes

**Fig. 2.**
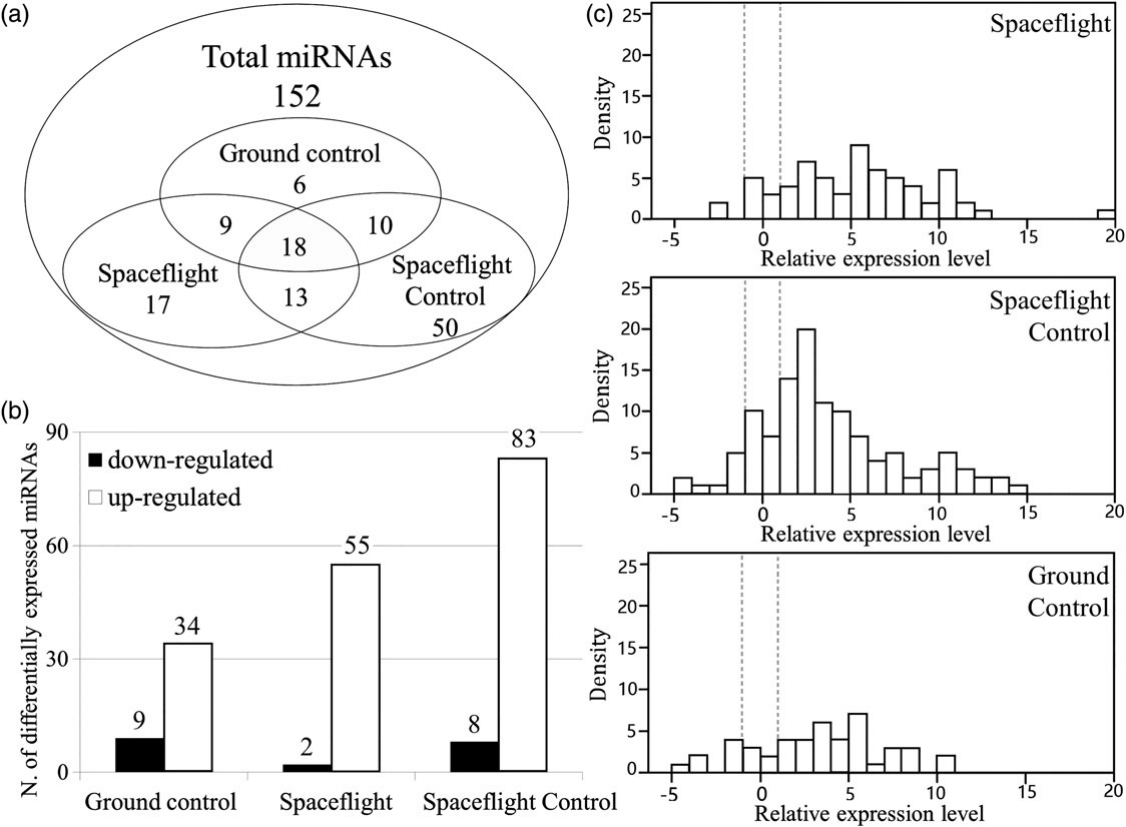
Analysis of miRNA profiling of *ced-1* mutant *C. elegans* under different conditions. (**a**) Venn diagram depicts the numbers of miRNAs being significantly affected by spaceflight (SF), spaceflight control (SC) and ground control (GC), with overlaps apparent among these groups. (**b**) The number of upregulated and downregulated miRNAs in each condition. (**c**) Distribution frequency of the 152 miRNAs affected by *ced-1* mutation in different conditions.

miRNA expression profiling was carried out in the *ced-1* mutant strain vs wild-type that underwent spaceflight (SF) condition, spaceflight control (SC) condition and ground control (GC) condition. The results showed that 43 out of 152 miRNAs in the microarray were differentially expressed in the GC condition, and the number of differentially expressed miRNAs increased to 57 and 91 in SF and SC conditions, respectively (Fig. 2a). Greater than 80% of the altered miRNAs were upregulated in each condition (Fig. 2b and c). The distribution frequency of the miRNA expression profiling indicated that space conditions differentially enhanced the number and the significance of the expression level affected by the *ced-1* mutation in ground samples, while microgravity combined with space radiation decreased them compared with spaceflight 1g incubation (Fig. 2c).

**Fig. 3.**
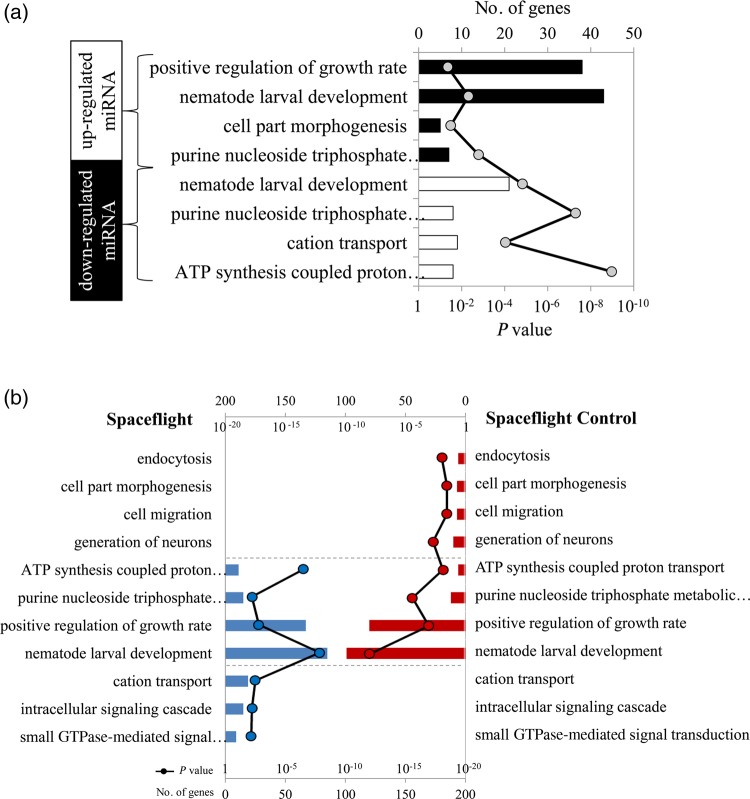
GO analysis of putative target genes regulated by altered miRNAs. (**a**) The biological processes affected by upregulated miRNA (white) and downregulated miRNA (black) in ground group. (**b**) The biological processes regulated by altered miRNAs in spaceflight group (blue) and spaceflight control group (red).

To analyze the biological processes regulated by miRNA, we predicted the target genes of differentially expressed miRNAs and determined the most enriched biological processes by GO analysis in relation to the different conditions. The results showed that nematode larval development was the most enriched biological process in each condition, and other processes differed depending on the conditions. In GC conditions, with the exception of nematode larval development and purine nucleoside triphosphate metabolic processes, genes involved in cation transport and ATP synthesis coupled with proton transport were regulated by downregulated miRNAs, and positive regulation of growth rate and cell part morphogenesis was regulated by upregulated miRNAs (Fig. 3a). There were four biological processes, including nematode larval development common to SF and SC conditions (Fig. 3b), small GTPase-mediated signal transduction, cation transcription and intracellular signaling cascades, which were enriched only in SF conditions. In SC conditions, enrichment occurred in categories of neuronal generation, cell migration, cell part morphogenesis and endocytosis.

### Integrated analysis of miRNA and genes involved in apoptosis

**Fig. 4.**
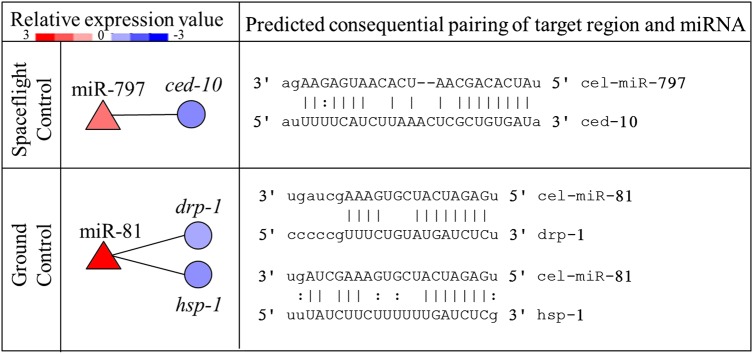
Integrated analysis of differentially expressed miRNA and target genes involved in apoptosis. The left column shows relative expression level of altered miRNA (red triangle) and the anti-correlated target genes (blue circle), and the right shows the predicted consequential pairing of target gene region and miRNAs.

In order to investigate the effects of spaceflight on apoptosis transcriptionally, we predicted the miRNAs involved in apoptosis and investigated known apoptotic gene expression profiling. By sequence similarity comparison with human miRNAs, 17 differentially expressed miRNAs related to apoptosis were identified (Table 2). By integrated analysis of these 17 miRNA and putative target genes, we found that miR-81 and 797 were anti-correlated to apoptotic genes *drp-1, hsp-1* and *ced-10* (Fig. 4).

**Table 2:** Analysis of sequence similarity of miRNA involved in apoptosis between *C.elegans* and *Homo sapiens*

cel-miR	Change fold (l*og 1.5*)			hsa- miR	Seq. blast (5′-3′, top-cel, bottom-hsa)	Pairing%
	SF	SC	GC			
miR-1	7.69	/	/	miR-1	UGGAAUGUaaagaaguaugua UGGAAUGUaaagaaguauguau	100
miR-256	5.12	2.23	−4.76	miR-1	UGGAAUGCauagaagacugua UGGAAUGUaaagaaguauguau	81.0
miR-796	2.30	−0.52	−1.98	miR-1	UGGAAUGUaguugagguuaguaa UGGAAUGUaaagaaguauguau	69.6
miR-48	/	13.83	/	let-7c	UGAGGUAGgcucaguagaugcga UGAGGUAGuagguuguaugguu	47.8
miR-84	3.11	7.00	3.21	let-7c	UGAGGUAGuauguaauauuguaga UGAGGUAGuagguuguaugguu	75.0
miR-795	−2.19	5.13	1.95	let-7c	UGAGGUAGauugaucagcgagcuu UGAGGUAGuagguuguaugguu	41.6
miR-81	10.31	6.87	10.04	miR-143	UGAGAUCAucgugaaagcuagu UGAGAUGAagcacuguagcuc	63.6
miR-82	11.95	11.62	7.96	miR-143	UGAGAUCAucgugaaagccagu UGAGAUGAagcacuguagcuc	54.5
miR-787	0.22	−3.84	−1.42	miR-99a	UAAGCUCGuuuuaguaucuuucg CAAGCUCGcuucuaugggucug	60.9
miR-73	/	7.06	7.65	miR-31	UGGCAAGAuguaggcaguucagu AGGCAAGAugcuggcauagcu	65.2
miR-235	/	−1.30	8.63	miR-92a	UAUUGCACucuccccggccuga UAUUGCACuugucccggccugu	81.8
miR-34	/	7.42	/	miR-34	AGGCAGUGugguuagcugguug UGGCAGUGucuuagcugguugu	86.4
miR-791	6.39	/	/	miR-96	UUUGGCACuccgcagauaaggcaa UUUGGCACuagcacauuuuugcu	58.3
miR-83	7.40	6.72	5.53	miR-29a	UAGCACCAuauaaauucaguaa UAGCACCAucugaaaucgguua	68.1
miR-90	4.99	6.54	5.14	miR-190	UGAUAUGUuguuugaaugccccu UGAUAUGUuugauauauuaggu	60.9
miR-124	1.40	0.17	−1.15	miR-124	GCAUGCACccuagugacuuuagu UAAGGCACgcggugaaugcc	47.8
miR-797	/	1.63	/	miR-499	/	^a^

cel = *C. elegans*, hsa = *Homo sapiens*, Seq. = mature miRNA sequence, SF = spaceflight, SC = spaceflight control, GC = ground control, Pairing = the number of matched base in mature cel-miRNA/the total number of base in mature cel-miRNA, ‘/’ = The low intensity differentially expressed miRNAs are filtered though median normalization method. The seed sequence are capitalized. ^a^ cel-miR-797 and hsa-miR-499 were identified with 5′ end sequence homology [48].

**Table 3:** The genes expression of core apoptotic pathway in different conditions

Gene	Description	Ensembl ID	Change fold (*log 2*)				
			SF		SC		GC
**Decision**							
*eor-1*	*egl-1* suppressor/ DiO uptake defective/ Raf enhancer, BTB /zinc-finger transcription factor	R11E3.6	−0.39		0.18		−0.16
*eor-2*	*egl-1* suppressor/ DiO uptake defective/ Raf enhancer	C44H4.7	**1.09**	**↑**	0.00		−0.35
*tra-1*	Transformer, repress *egl-1* to inhibit neurons apoptosis	Y47D3A.6	**0.08**	**↑**	**1.55**	**↑**	−1.09
*cep-1*	*C. elegans* P53-like protein, an ortholog of *p53*	F52B5.5	**0.14**	**↑**	**0.95**	**↑**	−0.82
**Execution**							
*ced-3*	Cell death abnormality, a cysteine-aspartate caspase,	C48D1.2	0.46		−0.31		0.39
*ced-4*	Cell death abnormality, adaptor	C35D10.9	**−0.21**	**↓**	**−0.33**	**↓**	1.47
*ced-9*	Cell death abnormality, cell-death inhibitor	T07C4.8	−0.56		**0.80**	**↑**	−1.08
**Engulfment**							
*ced-1*	Cell death abnormality, transmembrane protein for phagocytosis	Y47H9C.4	−0.37		**0.38**	**↑**	−1.18
*ced-2*	Cell death abnormality, adaptor for phagocytosis	Y41D4B.13	−0.03		−0.20		−0.87
*ced-5*	Cell death abnormality, scaffolding protein	C02F4.1	0.32		−0.26		−0.34
*ced-6*	Cell death abnormality, transmembrane protein for phagocytosis	F56D2.7	0.18		−0.22		0.43
*ced-7*	Cell death abnormality, transporter protein	C48B4.4	−0.74		**0.18**	**↑**	−0.84
*ced-10*	Cell death abnormality, GTPase for phagocytosis	C09G12.8	**−0.14**	**↑**	−1.23		−1.40
*ced-12*	Cell death abnormality, PH-domian protein	Y106G6E.5	−0.30		0.26		0.00
*pat-2*	Paralysed arrest at two- fold, alpha integrin subunit	F54F2.1	−0.27		−0.13		0.65
*pat-3*	Paralysed arrest at two- fold, beta-integrin subunit	ZK1058.2	0.07		0.18		0.03
*cdc-42*	Cell division cycle related, Rho GTPase	R07G3.1	**−0.17**	**↑**	**0.14**	**↑**	−2.02
*mig-2*	Abnormal cell migration, Rho family of GTP-binding proteins	C35C5.4	−0.05		−0.28		−0.01
*unc-73*	Uncoordinated, guanine nucleotide exchange factor	F55C7.7	**0.12**	**↑**	**0.18**	**↑**	−1.15
*nex-1*	An nexin family, phospholipid binding proteins	ZC155.1	−0.13		0.25		−0.46
*psr-1*	Phosphatidylserine Receptor family	F29B9.4	0.06		−0.07		0.54
*dyn-1*	Dynamin related	C02C6.1	−0.37		0.13		−0.06
**DNA degradation**							
*nuc-1*	Abnormal Nuclease, DNase II	C07B5.5	−0.21		−0.38		−0.18
*cps-6*	CED-3 protease suppressor, mitochondrial endonuclease	C41D11.8	−0.25		0.06		−0.93
*wah-1*	Worm AIF homolog, oxidoreductase	Y56A3A.32	−0.01		0.02		0.21
*crn-1*	Cell-death-related nuclease, 5′-3' exonuclease and endonuclease activity	Y47G6A.8	0.12		0.19		−0.57

DiO= diet-induced obesity, Raf = Rapidly Accelerated Fibrosarcoma, BTB= Broad complex, Tramtrack and Bric-a-Brac, GLI= glioblastoma, AIF = apoptosis inducing factor, SF= spaceflight, SC= spaceflight control, GC= ground control.

Gene was upregulated (**↑**) induced by SF/ SC condition when ( change fold _SF/SC_- change fold _GC_) >1; gene was downregulated(**↓**) when (change fold _SF/SC_- change fold _GC_) < 1.

### Analysis of apoptotic gene expression

Gene expression profiling of core apoptotic machinery showed that 10 of 26 genes functioning at different stages in the apoptotic pathway were differentially expressed, including *ced-4*, *ced-9*, *ced-1*, *ced-10*, *cdc-42*, *tra-1* and *unc-73* in GC samples, *eor-2* in the SF samples, and *tra-1* and *ced-10* in the SC samples (Table 3). Importantly, space conditions altered the expression value of genes in ground condition. According to the difference in the relative gene expression between space conditions and ground condition (difference value ≥1), seven genes were altered in SF condition, and eight genes in SC condition. Except for *ced-4*, most of the altered genes were upregulated by SF or SC conditions, indicating that space conditions could alter apoptosis on transcription.

### Verification of microarray expression levels by qRT-PCR

The microarray data from miRNA expression profiling was validated by qRT-PCR for five miRNAs, including miR-55 (GC), miR-56 (SC), miR-73 (SC), miR-84 (SF) and miR-124 (SF) (Table 4). From the independent worm samples used in the microarray, the qRT-PCR experiment showed that miR-84 and 73 were upregulated (>1.5-fold change), respectively; miR-55, 56 and 124 were unchanged (1.5 >fold change >0.67). The gene expression profiling was also validated by qRT-PCR, as carried out in our previous study [38].

**Table 4.** Validation of microarray results by using qRT-PCR

miRNA	Primer (5′→3′)	Group	Relative expression (mean ± *sd*)	
			microarray	qRT-PCR
U6	F:GGAACAATACAGAGAAGATTAGCA	SF/SC/GC	1	1
cel-miR-55	GSP:GCCCATACCCGTATAAGTTTCT	GC	1.55	0.91 ± 0.12
cel-miR-56	GSP:GCCACTACCCGTAATGTTTCC	SC	2.45	1.02 ± 0.09
cel-miR-73	GSP:GGTAAGGCACGCGGTGA	SC	17.53	2.43 ± 0.64
cel-miR-84	GSP:GGGGGGTGAGGTAGTATGTAAT	SF	3.53	2.53 ± 1.17
ced-miR-124	GSP:GGATGGCAAGATGTAGGCAG	SF	1.77	0.75 ± 0.32

GSP = gene specific primer, SF = spaceflight, SC = spaceflight control, GC = ground control.

## DISCUSSION

Whether space radiation and microgravity activate miRNAs to regulate apoptosis *in vivo* during spaceflight is undetermined. In the present study, we analyzed the differences in miRNA and core apoptotic gene expression profiles between wild-type and *ced-1* mutant *C. elegans* in response to space radiation and/or microgravity, by investigating dauer *C. elegans* under spaceflight, spaceflight 1g-control and ground control conditions during a 16.5-day spaceflight.

Previous studies have shown that worms can mate, reproduce, develop and undergo radiation-induced mutations during spaceflight experiments [32, 49]. In the present study, the morphology of wild-type and *ced-1* mutant space-flown worms was not changed (Fig. 1). This is likely due to the resistance of somatic cells to apoptosis, both under normal developmental conditions and during radiation [12, 50], as well as to the high resistance of dauer larvae to ROS damage and genomic instability [41–43]. However, the miRNA expression profiling showed differences between the *ced-1* mutant strain and the wild-type in GC condition, and space conditions increased the number and significance of differentially expressed miRNAs (Fig. 2). The altered miRNA regulated nematode development, growth and energy metabolic process in each condition (Fig. 3). These results are consistent with results from previous gene array studies on spaceflight using human lymphoblastoid cells [17], mice [51] and *C. elegans* [33], indicating metabolic and developmental perturbations may be conserved to assess the risk induced by spaceflight [33].

Similar to the results reported for irradiated human lymphocytes [24] and our previous study using wild-type *C. elegans* [38] (Supplementary Table 2), we found that microgravity decreased the number of miRNAs when compared with 1 g condition in the presence of radiation, and it is speculated to induce different categories of biological processes between SF and SC conditions. In the SF samples, most of the target genes enriched in intracellular signaling cascades were involved in small GTPase-mediated signal transduction. While in the SC samples, miRNA regulated endocytosis, cell migration and neurons compared with the response in GC samples (Supplementary Table 1). Small GTPases, including Ras, Rho, Rab, ARF and Ran, are a family of hydrolase enzymes that can bind and hydrolyze GTP and can determine the temporal aspects of a broad variety of signaling events involved in numerous cellular processes and responses [52]. Studies have shown that both microgravity and radiation modulated the cytoskeleton by regulating the activation of Rho [53–55]; Ras-related small GTPases regulated apoptosis and DNA repair in an irradiated cell line and in irradiated *C. elegans* [56, 57]. CED-10 within an engulfing cell is also a small GTPase [58]. Therefore, small GTPase signaling transduction may be activated by miRNA in response to SF condition, including space radiation and microgravity stress. The results in the SC samples mainly relate to the apoptotic process, suggesting that apoptosis plays a vital role in neurogenesis during development [59], and that being exposed to space radiation may enhance the impact resulting from engulfment defects in the *ced-1* mutant *C. elegans* [46, 60]. In general, we speculate that microgravity could alter the miRNA expression signature [24, 29] and the regulation of small GTPase-mediated signal transduction when compared with 1 g incubation in the presence of space radiation.

Focusing on apoptosis, we first screened 17 altered miRNAs related to apoptosis by comparison to the sequence similarity of human apoptotic miRNAs, since core apoptotic pathways and most miRNAs are well conserved from *C. elegans* to human beings [61, 62]. As shown in Table 2, the obvious differences in the miRNA expression signature between the three groups indicates that spaceflight might regulate apoptosis at the post-transcriptional level, and that microgravity may be involved in the apoptotic process, combined with space radiation. Worth noting is that cel-miR-1/256/796, cel-miR-48/84/795 and cel-miR-81/82 have high homology to hsa-miR-1, hsa-let-7 and hsa-miR-143, which have been demonstrated to function in apoptosis in response to IR or microgravity [63–68]. Other human miRNAs having sequence similarity to *C. elegans* were also proven to be involved either directly or indirectly in apoptosis [39, 69–72]. In order to refine the functional miRNA–mRNA relationship, we performed integrated analysis of the miRNA and mRNA expression profiles and found two pairs of significantly anti-correlated miRNA and apoptotic genes, including miR-797 and *ced-10* in SC conditions and miR-81 and *drp-1*, *hsp-1* in GC conditions (Fig. 4). cel-miR-797 has a high sequence similarity to hsa-miR-499 [48], which could protect cardiomyocytes from H_2_O_2_-induced apoptosis and regulate the expression of the tumor suppressors Forkhead box O 4 (FOXO4) and programmed cell death 4 (PDCD4) [69, 73]; the target gene *ced-10* functions with *ced-2* and *-5* to promote formation of the polarized cell extensions associated with cell corpse engulfment during apoptosis [58]. cel-miR-81 may regulate apoptosis according to the sequence-similar hsa-miR-143, which has been shown to regulate the apoptotic process by affecting MAPK/Extracellular signal-regulated kinase (ERK) signaling transduction and the expression of Bcl-2, pro-caspase-3 and -9 [56, 57]; the target gene *drp-1*, encoding dynamin-related protein 1 (DRP-1), regulates distinct cell-death execution pathways downstream of *ced-3* and independent of ced-9 [74]; another target gene *hsp-1* encodes Hsp70A, which is involved in receptor-mediated endocytosis and apoptosis by GO identification. In addition, a previous study reported that the heat shock protein HSP72 accumulated in response to the space environment in goldfish [75]. Hence, we speculated that the apoptotic response to space radiation might be attributable to *ced-10*-mediated engulfment regulated by miR-797, while miR-81 might be involved in apoptosis by regulating *drp-1* and *hsp-1*.

It has been reported that checkpoints and physiological apoptosis in germ cells proceed normally in space-flown *C. elegans* by evaluating the expression of apoptotic execution and checkpoint genes [31]. Ground-based studies have reported that MMG inhibited apoptosis in irradiated lymphocyte cells by delaying double-strand break re-joining or amplifying ROS impacts [20, 21]. However, the transcriptional response of the integrated core apoptotic pathway to space conditions *in vivo* has not been documented. In our study, we found that space conditions could regulate various stages of apoptosis. Possibly due to the resistance to ROS and genomic instability in dauer larvae, there was little difference between the *ced-1* mutant and the wild-type larvae in space conditions. When compared with GC samples, space conditions both altered decision and execution of apoptosis and promoted engulfment transcriptionally (Table 3). Upregulated genes acting in the engulfment stage indicated that apoptotic engulfment were promoted in both space conditions, but genes in other stages showed different change manners. In SC condition, apoptosis was more likely to decrease according to the expression of execution genes *ced-4,* and *ced-9* and decision gene *tra-1*, although the pro-apoptotic gene cep-1 was upregualted. Alteration tendency of apoptosis in SF condition was not determined, because the opposing regulation by *cep-1*, *eor-2*, and *tra-1*. It is speculated that these changes on apoptosis result from interaction of environmental factors and genetic factors. Ground-based study has shown that apoptosis is reduced by protons but induced by γ-rays at the gene expression level during long-term spaceflight [76], indicating that the composition of complex types of space radiation may contribute to the perturbation of apoptosis. Environmentally, although the dose of space radiation is relative low in our study, the effect of space radiation can't be denied, because space radiation has been proven to induce obvious effects, even though the dose is three orders of magnitude lower than that of ground-based simulation [36]. Microgravity also may contribute to the complex regulation of apoptosis in the presence of space radiation [21, 22]. Genetically, combined with the changes in miRNA, increased engulfment, affected by miR-797, may help protect against the abnormal apoptosis induced by space conditions. The results suggest that there may be a feedback loop for the apoptotic process to promote cell survival, supplied by miRNAs and apoptotic genes [39, 40], e.g. miR-81, or by the different stages of apoptosis, e.g. decision, execution and engulfment. Further study on the function of miRNAs and target genes during apoptosis will be performed. In generally, apoptosis might undergo an adaptive modification at the transcription level in response to space conditions [77], and miRNA could be involved in different stages of the process.

In conclusion, miRNAs are probably involved in apoptosis during short-term spaceflight. Space conditions increase changes in the expression signature of miRNAs that could affect development, energy metabolism, apoptosis and signaling transduction in *C. elegans*. In comparable 1g incubation in the presence of space radiation, microgravity decreases the number and significance of differentially expressed miRNAs, and regulates the expression signature of potential apoptotic miRNAs and genes acting in the decision, execution and engulfment stages in the core apoptotic pathway.

## SUPPLEMENTARY DATA

Supplementary data are available at the *Journal of Radiation Research online*.

## FUNDING

This work was supported by the National Natural Science Foundation of China (No. 31270903) and Fundamental Research Funds for the Central Universities (No. 3132014306). Funding to pay the Open Access publication charges for this article was provided by the National Natural Science Foundation of China (No.31270903).

## Supplementary Material

Supplementary Data
