# Supplementary material for: Changes in apoptotic microRNA and mRNA expression profiling in Caenorhabditis elegans during the Shenzhou-8 mission
Source: J Radiat Res. 2015 Aug 17;56(6):872–82. doi: 10.1093/jrr/rrv050 (PMC4628221; doi:10.1093/jrr/rrv050)
Supplement: Supplementary Data [file supp_56_6_872__index.html]

Changes in apoptotic microRNA and mRNA expression profiling in Caenorhabditis elegans during the Shenzhou-8 mission — Changes in apoptotic microRNA and mRNA expression profiling in Caenorhabditis elegans during the Shenzhou-8 mission — Supplementary Data 

# Changes in apoptotic microRNA and mRNA expression profiling in *Caenorhabditis elegans* during the Shenzhou-8 mission

## Supplementary Data

Supplementary Data

- Supplementary Data - Docx file
