# Supplementary material for: Changes in apoptotic microRNA and mRNA expression profiling in Caenorhabditis elegans during the Shenzhou-8 mission
Source: J Radiat Res. 2015 Aug 17;56(6):872–82. doi: 10.1093/jrr/rrv050 (PMC4628221; doi:10.1093/jrr/rrv050)
Supplement: Supplementary Data [file supp_rrv050_rrv050supp.docx]

Supplementary Table 1 Biological processes of putative target genes enriched in under different conditions

| GO. ID | GO term | Gene |
| --- | --- | --- |
| **Ground Control up-regulated** | |  |
| GO:0040010 | Positive regulation of growth rate | *daf-9,* B0336.3*, tir-1, rgl-1, sax-3, mca-3, vha-2, dpy-2, hgrs-1, paa-1,* B0416.5, *unc-130, unc-115, mca-1, cgh-1,* C15C8.4*, daf-12, scd-1, gld-2, eft-4, ncx-2, sem-4,* C13F10.4*, ketn-1, peb-1, dbl-1, osm-11, sel-10, vha-13, jun-1,* F13H6.1, T03F1.8, *pdi-2, din-1, unc-52, snr-1,* F14E5.2*, rpl-33* |
| GO:0002119 | Nematode larval development | *lin-1, glf-1, daf-9, tir-1, mlc-2, pmk-2, ptc-1, ptr-3, hsp-1, mca-3, gpb-1, hgrs-1, vha-2, pat-2, paa-1, eat-6, daf-16, vha-12, cgh-1, sma-5, cul-1, let-805, eft-4, rskn-2, dsh-1,* C13F10.4*, peb-1, ifb-1, dbl-1, syd-9, osm-11, vha-13, hmr-1, jun-1, pdi-2, din-1, unc-52, snr-1, rpl-33, hbl-1, col-43, ace-3, rab-11.1* |
| GO:0032990 | Cell part morphogenesis | *lrk-1, zag-1, crml-1, unc-130, drp-1* |
| GO:0009144 | Purine nucleoside triphosphate metabolic process | *eat-6, vha-12, mca-1, vha-13, vha-2, mca-3,* F55F3.3 |
| **Ground Control down-regulated** | |  |
| GO:0002119 | Nematode larval development | C08H9.2*,* F32D1.2*, ptc-1, vha-8, vha-9, fbn-1,* F49C12.12, *vha-13, vha-14, pat-2, vha-17, vha-1, unc-112,* C10E2.6, *jun-1, erm-1, daf-16, vha-12, unc-52, ace-3, lir-1* |
| GO:0006812 | Cation transport | F32D1.2*, vha-8, vha-12, vha-9, vha-13, vha-14, vha-17, vha-1, nac-3* |
| GO:0015986 | ATP synthesis coupled proton transport | F32D1.2*, vha-8, vha-12, vha-9, vha-13, vha-14, vha-17, vha-11* |
| GO:0040010 | Purine nucleoside triphosphate metabolic process | F32D1.2*, vha-8, vha-12, vha-9, vha-13, vha-14, vha-17, vha-1* |
| **Spaceflight** | |  |
| GO:0006812 | Cation transport | F32D1.2, *vha-8, vha-9, vha-13, vha-14, vha-2, mca-3, vha-16, vha-17, vha-1, nac-3, eat-6, vha-12, vha-11, f27c1.2, mca-1,* T21C9.3, F55F3.3, *ncx-2* |
| GO:0007242 | Intracellular signaling cascade | r*gl-1, rab-1, pac-1, pmk-2, lrk-1*, C43G2.1, F35H12.4, *pkc-1,* F52A8.6, *pxf-1, rskn-2, ran-1, mpk-1, rab-11.1, dsh-1* |
| GO:0007264 | Small GTPase mediated signal transduction | *rgl-1, rab-1, pac-1*, *lrk-1,* F52A8.6, *pxf-1, ran-1, mpk-1, rab-11.1* |
| **Spaceflight Control** | | |
| GO:0048699 | Generation of neurons | *mup-2, lrk-1, zag-1, lin-23, mig-15, unc-130, hda-4, unc-70, egl-44, sax-1* |
| GO:0032990 | Cell part morphogenesis | *mup-2, lrk-1, zag-1, lin-23, mig-15, unc-130, drp-1* |
| GO:0006897 | Endocytosis | *ced-5, rme-1, ced-7, lrp-1, mca-3, rab-11.1* |
| GO:0016477 | Cell migration | *ced-5, unc-52, lrp-1, dre-1, hmr-1, mig-15, ceh-18* |

Supplementary Table 2 Differentially expressed miRNAs in spaceflight groups versus ground group of wild type *C.elegans*

| miRNA | Change fold (*log 1.5*) | |
| --- | --- | --- |
|  | Spaceflight group/ Ground group | Spaceflight control group/ Ground group |
| cel-miR-256 | -6.57 | -7.59 |
| cel-miR-124 | -1.72 | -1.53 |
| cel-miR-796 | -2.05 | -1.71 |
| cel-miR-84 | -1.40 | -2.39 |
| cel-miR-82 | -5.09 | -4.18 |
| cel-miR-56 | 1.43 | 1.71 |
| cel-miR-237 | 1.47 | 2.12 |
| cel-miR-81 | -3.79 | - |
| cel-miR-265 | -3.49 | - |
| cel-miR-1823 | -1.36 | - |
| cel-miR-52 | 1.44 | - |
| cel-miR-1822 | 2.63 | - |
| cel-miR-257 | 1.37 | - |
| cel-miR-795 | 2.61 | - |
| cel-miR-230 | - | -1.60 |
| cel-miR-258 | - | -1.98 |
| cel-miR-73 | - | 1.52 |
| cel-miR-55 | - | 1.59 |
| cel-miR-788 | - | 2.69 |
| cel-miR-235 | - | 7.89 |
| cel-miR-799 | - | 1.71 |
| cel-miR-1824 | - | 2.43 |
| cel-miR-787 | - | 1.09 |
